# Supplementary figures and images for: Implementation evaluation of a medical student-led intervention to enhance students’ engagement with research: Findings and lessons learned
Source: PLoS One. 2023 Aug 31;18(8):e0290867. doi: 10.1371/journal.pone.0290867 (PMC10470873; doi:10.1371/journal.pone.0290867)

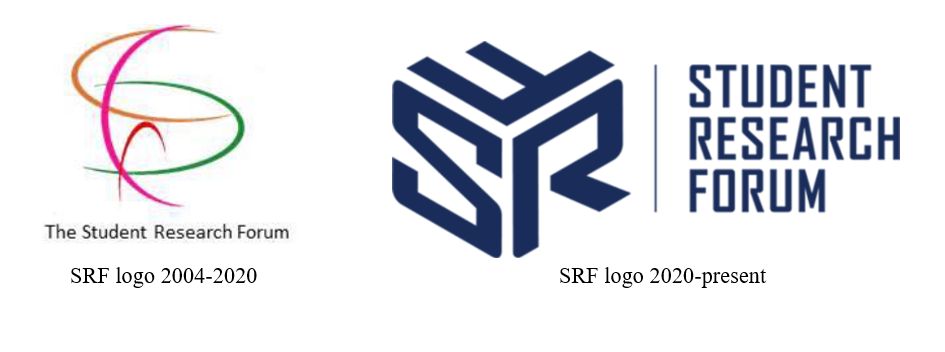

Supplement: S1 Fig — (TIF) [file pone.0290867.s001.tif]
