# Supplementary material for: Implementation evaluation of a medical student-led intervention to enhance students’ engagement with research: Findings and lessons learned
Source: PLoS One. 2023 Aug 31;18(8):e0290867. doi: 10.1371/journal.pone.0290867 (PMC10470873; doi:10.1371/journal.pone.0290867)
Supplement: S1 Table — (PDF) [file pone.0290867.s002.pdf]

S1 Table. SRF events and activities held during the study duration

Date	Event	Theme	Participants	Objectives & Description	Wings and Collaborations
April 2019	STATA 101[1]	Research Skills	100[a]	Students learned how to use the research software STATA and practiced on datasets shared by the speaker.	Workshops
April 2019	Meet the Matched-2019[1]	Mentorship	300[a]	Medical students met with recent graduates who have recently been accepted into residencies in the US and acknowledged the importance of research in their journeys.	Flagship event
April 2019	The Roundtable A Journal Club Series: Episode 2[1]	Cardiothoracic Surgery	60[a]	20 students published case reports and 4 students published full length articles, with several others under journal review. 9 reports were presented at CHEST 2020.	Academics
May 2019	Case Report Writing 101[1]	Research Skills	35[a]	Medical students were taught about the ins and outs of case report writing. Such sessions also aim to foster PGME-UGME relationships.	Resource Mentorship and Development
July 2019	The Roundtable A Journal Club Series: Episode 3[1]	General Surgery	69[a]	Participants were informed how the speaker created a method to audit surgery costs and its use. Participants then formulated novel research ideas.	Academics
August 2019	The Roundtable A Journal Club Series: Episode 4[1]	Trauma Surgery	65[a]	Participants discussed inequities in surgery and the significance of equitable outcomes in trauma surgery and surgical academia.	Academics

August 2019	Women in Research[1]	Mentorship: neurology, cardiology, endocrinology, psychiatry, community health sciences, biomedical sciences	60[a]	Discussed the importance of research from a physician-scientist's perspective, the struggles faced during their research journeys, and how individuals and organizations can come together to find solutions to these challenges.	Resource mentorship and development
November 2019	1st Chai and Chat[1]	Mentorship: General surgery and pediatrics	Open house event - estimated 50[b]	Small group discussions and one on one conversations with faculty about research ideas and careers as academic professionals in medicine.	Resource mentorship and development
December 2019	The Roundtable A Journal Club Series: Episode 5[1]	Pulmonology	40[a]	Students discussed the speaker's research topics and were informed of the value of and lessons learned from presenting at research conferences. Four participants availed research opportunities with the speaker and projects are currently underway.	Academics
December 2019	2nd Chai and Chat[1]	Thoracic surgery, cardiology	79[b]	A two part event consisting of a presentation followed by an interactive session. Students discussed the significance of research in their academic careers, challenges they may encounter, and how to overcome them.	Resource mentorship and development, collaboration with Surgery Interest Group
January 2020	The New Era of Research: Data Science in Health[1]	Data Science	40[a]	The audience was introduced to the role of data science in transforming the landscape of medicine.	Mentorship and Development
Januar	The	Cardiology	31[a]	The speaker dissected his	Academics

y 2019	Roundtable A Journal Club Series: Episode 6[1]			study on Treatment QI in LMICs & CT scan radiation exposure in patients	
February 2020	The Roundtable A Journal Club Series: Episode 7[1]	Neurology	40+[a]	Participants were introduced to critical appraisal of evidence based literature.	Academics
February 2020	The Research Games[1]	Research Skills	32[a]	Students were taught how to design research questions.	Workshops
April 2020	Meet the Matched 2020[1]	Mentorship	300+[b]	Medical students met recent graduates who had recently been accepted into residencies in the US and acknowledged the importance of research in their journeys.	Flagship event
May 2020	The Roundtable A Journal Club Series: Episode 8[1]	Infectious Disease	100+[b]	The latest COVID-19 research and strategies to stem the pandemic were discussed by the country's leading COVID-19 expert.	Academics
May 2020	The Roundtable A Journal Club Series: Episode 9[1]	Cardiology	66[a]	The speaker dissected his study on Thrombolysis in Pulmonary Embolism and spoke in depth about how meta-analysis can be used to critically appraise clinical data.	Academics
May 2020	What happened to my paycheck?[1]	Mentorship: Financial management	120+[b]	The audience gained an idea about initial earnings, potential expenses, insurance plans & the importance of savings during residency in the US.	Records & Finance
May 2020	The Roundtable A Journal Club Series: Episode 10[1]	Neurosurgery	70+[a]	Participants learned about the role of neurosurgery in global surgery & the role of mRNA in tumor invasion.	Academics

May 2020	A Beginners Guide to Research[2]	Research Skills	3800+[b]	Nationwide workshop on introduction to research & medical ethics, conducting a literature search, the basics of abstract and manuscript writing, and the basics of referencing.	Workshops and Skill Development
July 2020	Research 101[2]	Research Skills	1200+[b]	Nationwide workshop on introduction to research & medical ethics, conducting a literature search, the basics of abstract and manuscript writing, and the basics of referencing.	Workshops & Skills Development
July 2020	Anki 101: A Beginner's Guide on How Not to Forget Stuff[1]	Medical Education	80+[a]	Attendees were taught how to use the Anki application effectively & maximize its use.	Collaboration between Academics and Mentorship and Resource Development
July2020	The Roundtable A Journal Club Series: Episode 11[1]	Research Skills and Pediatrics	65[a]	The audience was taught how to measure the reliability, accuracy & applicability of research.	Academics
July 2020	SRF Alumni Series Episode 1: Meet the Experts[1]	Mentorship	40+[a]	The speakers engaged the audience in a discussion on research & residency during the COVID-19 pandemic.	Mentorship and Development
September 2020	SRF Alumni Series Episode 2[1]	Public Health	30+[a]	The speaker gave the audience an overview of his experiences in Public Health Research.	Mentorship and Development
September 2020	SRF Alumni Series Episode 3[1]	Research Skills	30+[a]	Participants learned how to write manuscripts & edit research papers.	Mentorship and Development
March 2021	The Roundtable:	Trauma Surgery and	40+[a]	The speaker spoke about his paper on the	Academics

	A Journal Club Series Episode 12[1]	Sepsis		significance of histone deacetylase inhibitors in decreasing mortality from traumatic brain injury and post-doctoral research in the US.	
April 2021	Inventing the World's First Portable MRI: The Role of Research in Industry[2]	Biomedical Engineering	60+[a]	The audience was introduced to the groundbreaking implications of portable medical imaging techniques on the future of medicine.	Records & Finance
April 2021	Meet the Matched 2021[1]	Mentorship	300+[b]	Medical students meet recent graduates who have recently been accepted into residencies in the US and acknowledge the importance of research in their journeys.	Mentorship and Development
April 2021	Live Demonstration: Hyperfine MRI Machine[1]	Biomedical Engineering	60+[a]	The role of a safe, accessible, portable MRI machine in healthcare was demonstrated in real time to participants.	Records & Finance
April 2021	Crossing Borders: An Insight to the UK Training Experience[2]	Mentorship	150+[b]	The audience was guided about the application process and the role of research for medical training in the UK.	Mentorship and Development
May 2021	From a Jester to An-King: Mastering the Basics of Anki[1]	Medical Education	60+[a]	Attendees were taught how to use the Anki application effectively & maximize its use.	Academics
May 2021	Research & SRF: An Introduction[1]	Research Skills	50+[a]	The audience was taught how to perform data collection followed by an interactive chat with	Workshops

				research mentors.	
May 2021	Personal Financial Management During Residency in the US[1]	Mentorship: Financial management	85[a]	The audience gained an idea about initial earnings, potential expenses, insurance plans & the importance of savings during residency in the US.	Records & FInance
May 2021	Med School and Beyond: Insta Live with SRF[2]	Mentorship	130[b]	Recent graduates shared their experiences on research, networking, interviews, and resume writing.	Communications
June 2021	The Roundtable: A Journal Club Series Episode 13[2]	Psychiatry	60+[a]	Students discussed the factors affecting suicide in Karachi, Pakistan and research on neglected topics.	Academics
June 2021	From Squires to Knights[1]	Mentorship and Research Skills	104[a]	The audience was informed on how to pursue their own research pathways.	Academics and Communications
July 2021	The Roundtable: A Journal Club Series Episode 14[1]	Gastroenterology	35[a]	The audience learned about video case reports, how to prepare them & their relevance in a digital world.	Academics
July 2021	Mentorship From Seniors[2]	Mentorship	130+[b]	Students learned how appropriately & professionally seek out & approach alumni and seniors.	Communications
August 2021	Stats with SRF[1]	Research Skills	40[a]	The basics of SPSS & common parametric tests were taught to the audience.	Workshops
September 2021	Meta Analysis: Everything You Need To Know[2]	Research Skills	50[a]	The participants gained a basic understanding of systematic reviews & meta-analysis	Workshops

1=Open to home institute (AKU) participants only, 2=Open to all participants irrespective of institute, a=All participation spots filled, b=Unlimited participation spots available
