# Supplementary material for: Implementation evaluation of a medical student-led intervention to enhance students’ engagement with research: Findings and lessons learned
Source: PLoS One. 2023 Aug 31;18(8):e0290867. doi: 10.1371/journal.pone.0290867 (PMC10470873; doi:10.1371/journal.pone.0290867)
Supplement: S2 Table — (PDF) [file pone.0290867.s003.pdf]

S2 Table. List of Student Ambassador Program Ambassadors' Institutes

Institute	Program	City
**Azad Jammu and Kashmir (Administrative Region)**		
Mohtarma Benazir Bhutto Shaheed Medical College	MBBS	New Mirpur
**Balochistan (Province)**		
Bolan University of Medical and Health Sciences	MBBS	Quetta
**Islamabad Capital Territory**		
Al Nafees Medical College	MBBS	Islamabad
Foundation University and Medical College	MBBS	Islamabad
Margalla Institute of Health Sciences	BDS	Islamabad
Shifa Tameer-e-Millat University	BScN	Islamabad
	MBBS	Islamabad
Wah Medical College	MBBS	Islamabad
**Khyber Pakhtunkhwa (Province)**		
Institute of Management Sciences (IMSciences)	BBA (Healthcare Management)	Peshawar
Northwest School of Medicine	MBBS	Peshawar
**Punjab (Province)**		
Allama Iqbal Medical College	MBBS	Lahore
Army Medical College	BDS	Rawalpindi
	MBBS	Rawalpindi
CMH Lahore Medical And Dental College	DPT	Lahore
Fatima Jinnah Medical University	MBBS	Lahore
HBS Medical and Dental College	MBBS	Rawalpindi
Islamabad Medical and Dental College	MBBS	Rawalpindi

Islamic International Dental College	BDS	Rawalpindi
King Edward Medical University	MBBS	Lahore
Nishtar Medical University	MBBS	Multan
Rawalpindi Medical College	MBBS	Rawalpindi
Riphah International University (Faisalabad)	DPT	Faisalabad
Riphah International University (Islamabad)	BDS	Rawalpindi
	MS CPPT	Rawalpindi
Shalamar Medical & Dental College	MBBS	Lahore
University of Lahore	Pharm D	Lahore
**Sindh (Province)**		
Aga Khan University	MBBS	Karachi
	BScN	Karachi
Baqai Medical University	BS Bioinformatics	Karachi
	Pharm D	Karachi
Dow University of Health Sciences	BS Biotechnology	Karachi
	Pharm D	Karachi
	BDS	Karachi
	BScN	Karachi
	MBBS (Dow Medical College)	Karachi
	MBBS (Dow International Medical College)	Karachi
Horizon School of Nursing & Allied Health Sciences	BScN	Karachi
	Post RN BSN	Karachi
Indus College of Nursing & Midwifery	BScN	Karachi
Isra University	MBBS	Karachi
Jinnah Postgraduate Medical Center	DPT	Karachi
	Masters in Physiotherapy	Karachi
Jinnah Sindh Medical University	Pharm D	Karachi

	BBA	Karachi
	BScN	Karachi
	MBBS	Karachi
Jinnah University for Women	Pharm D	Karachi
Karachi Institute of Medical Sciences	MBBS	Karachi
Karachi Medical and Dental College	BDS	Karachi
	MBBS	Karachi
Karachi University	BS Microbiology	Karachi
Liaquat National Hospital and Medical College	BScN	Karachi
	MBBS	Karachi
Liaquat University of Medical and Health Sciences	DPT	Jamshoro
Memon College of Nursing	BSN	Karachi
Patel Institute of Nursing	BScN	Karachi
Saifee Burhani School of Nursing	BScN	Karachi
Savastu Institute of Nursing	BScN	Karachi
Shaheed Mohtarma Benazir Bhutto Medical College	MBBS	Karachi
Sindh Institute of Oral Health Sciences	BDS	Karachi
Sir Syed College of Medical Sciences for Girls	MBBS	Karachi
Ziauddin University	Post RN BSN	Karachi
	Pharm D	Karachi
	MBBS	Karachi
	BScN	Karachi
