# Supplementary material for: Implementation evaluation of a medical student-led intervention to enhance students’ engagement with research: Findings and lessons learned
Source: PLoS One. 2023 Aug 31;18(8):e0290867. doi: 10.1371/journal.pone.0290867 (PMC10470873; doi:10.1371/journal.pone.0290867)
Supplement: S1 File — (PDF) [file pone.0290867.s004.pdf]

S1 File. Description of Student Research Forum (SRF) Wings

Academics

SRF's academics wing focuses primarily on conducting academics related events such as journal clubs. The wing has been organizing journal clubs with national and international faculty to discuss research breakthroughs in the fields of medicine and healthcare.

Additionally, the academics wing organizes sessions that tie in with Academia such as on Anki, a memory learning application, to guide beginners about its basics as well as the advanced functions of the application to aid in their studies. The wing plans events such as "Healthy Skepticism" to highlight the drawbacks and problems associated with research. In order to promote student research leaders within, the wing introduced the series "From Squires to Knights" where different student researchers share their journeys and inspire the audience to start their own projects.

Communications

SRF's communications wing is the backbone of every activity arranged by SRF. It is responsible for handling social media, as well as its marketing and content. The wing's work ranges from making creative posters to writing content and handling the messages and queries received on SRF's social media accounts.

Social media marketing is an essential component of SRF as it is the first interaction the organization is able to make with its audience prior to attending our events. It is important for SRF to create a lasting impression using the communications wing's creative prowess and professional communication skills.

Records and Finance

The records and finance wing plays an indispensable role when it comes to keeping track of the events orchestrated by SRF. It is responsible for preparing a detailed report of all the events conducted by SRF, and these reports highlight the key points discussed in each event and also comment on interaction between the audience, the speakers, and the moderators. These reports therefore help SRF to reflect upon what has been done throughout the year in an efficient manner.

The records and finance wing has also been tasked with designing cost-effective plans and is currently in the process of doing so. Furthermore, the wing has worked in conjunction with other societies to organize a series of events. This has included collaborating with student research organizations in other institutes to provide experiential advice to replicate SRF's model. The records and finance wing's primary objective is to maximize SRF's efficiency in carrying out its activities by providing operational guidance and logistical support.

Research Development and Mentorship
The research development and mentorship (RDM) wing is an amalgamation of two of SRF's most principal pillars: research and mentorship. RDM integrates a network of AKU faculty and alumni, helping them connect with suitable AKU students for national and international research opportunities. RDM maintains a meticulously formulated and regularly updated database which matches students' interests and their proficiency in different research skills to the most desirable projects.

RDM is also responsible for arranging alumni talks with AKU graduates serving at top research and leadership positions in the US, Pakistan, and throughout the world. These intellectually stimulating conversations have been essential in shaping young minds to doctors of tomorrow. From choosing a specialty to making connections, and from preparing for residency interviews to finding guidance regarding every step of the medical school journey, SRF's mentorship program works to ensure that all students learn these lessons from those who have walked the same paths years ago.

Strategic Planning
The strategic planning wing is responsible for reviewing current events, processes, and structures within the organization and suggesting improvements to ensure that all the many wings work together cohesively. The wing is entrusted with brainstorming and delineating ideas and future trajectories that would allow SRF to remain at the forefront of student-led research in the country. The wing works closely with the records and finance wing to evaluate SRF's activities and concurrently design its future direction.

Workshop & Skills Development
The workshop and skills development wing furthers SRF's goal of helping medical students produce high quality and impactful research. It organizes workshops that help students start their research journeys by learning about foundational research skills such as manuscript and abstract writing, conducting literature searches, and utilizing statistical software. The wing reaches out to faculty members, alumni, and current SRF members who have extensive research and mentoring experiences to lead these sessions.

The wing organizes multiple workshop series in a year suited for different skill levels and audiences–some being exclusive to AKU students, while others are open to the national medical student community as well as high school students aspiring to join medical school. Furthermore, the workshop and skills development wing has organized interactive talks with alumni members to learn from their medical school and match experiences.
