# Supplementary material for: Implementation evaluation of a medical student-led intervention to enhance students’ engagement with research: Findings and lessons learned
Source: PLoS One. 2023 Aug 31;18(8):e0290867. doi: 10.1371/journal.pone.0290867 (PMC10470873; doi:10.1371/journal.pone.0290867)
Supplement: Attachment — Submitted filename: Plos One review.docx [file pone.0290867.s005.docx]

This study aims to describe how implementing a theory of change led to increasing student engagement with research. A theory of change is a description of how an intervention is intended to lead to outcomes for beneficiaries and changes the focus of concern from what is being *done*, to what is to be *achieved*. Once a theory of change is in place, it can be used to evaluate the outcomes and establish whether and how an initiative works. If failure of the theory is observed, then questions can be asked about whether that was due to the theory being adopted (i.e. whether the theory is flawed) or whether the interventions need to be changed. The study describes how interventions were put into place through the vehicle of a Student Research Forum to improve student engagement with research and then evaluated on key metrics of reach, adoption and efficacy. The study concludes that the reach of the Student Research Forum increased and efficacy was measured based on the number of research projects resulting from the engagement.

Recommendations

1. Ethics. The authors have indicated that no ethical statement was needed for this study. However, in any research or evaluative study there are ethical implications for how it is carried out, even if no formal ethical approval has been sought. I had a number of concerns relating to ethics in this paper, first and foremost that it is not considered at all in the text. In addition, participants names are used, as well as organisational and role details. In and of itself, this may not be an issue, providing that permission has been sought from participants, and any unintended consequences have been considered. If this is the case, then a paragraph needs to be inserted to explain this process and the resulting ethical decisions. If not, then I would suggest anonymising any data pertaining to individuals. I would recommend referring to the British Educational Research Association (BERA) guidelines in this process as a starting point.
2. The use of theory of change. The title implies that the paper explores the efficacy of the theory of change process. This however does not materialise and, rather, the paper explores the reach and efficacy of the interventions rather than the theory of change as an approach. I would recommend that either the title is changed to reflect this, or that more details are provided about the theory of change process itself. This needs to comprise a more detailed examination of literature concerning theory of change approaches situated in a consideration of alternative approaches that could be used. The paper then needs to explain the limitations of the approach as well as the potential benefits and provide an explanation in the conclusion about any challenges or successes that emerged from the theory of change approach.
3. Interventions. It is not clear to me how the theory of change process led to the implementation of particular interventions. I would recommend considering the evidence for particular interventions in your literature review and then explaining how the adoption of a theory of change approach led to the authors (and others?) deciding these were appropriate interventions.
4. The model. I recommend giving a description of how the theory of change approach was carried out. What kinds of activities were used to develop the theory of change? How was consensus reached? How did the theory of change help in deciding what to do?
5. Data analysis. ‘implementation outcomes were either adopted from existing literature or defined as novel and tailored interpretation’. This needs explaining and more detail given. What literature? How were they decided?
6. Results. These are results of the evaluation based on reach, implementation and efficacy, rather than the results of using a theory of change approach and this should be made clear, and the metrics for measuring reach, adoption and efficacy clearly outlined at the beginning of the section, or in previous section around methods. The methods need to state how this information was collected, what form it was in, how it was analysed and what permissions were sought to use it.
7. I remain unsure of how Figure 1 relates to the theory of change. The outcomes for students are listed as objectives, rather than steps in the theory to enhance student engagement with research. The intermediate outcomes seem more like a list of measurements than expected changes. I would recommend reading and referring to work by Carol Weiss, and also by Mackenzie and Blamey, and then finally by Connell and Klem. Pawson’s work also provides a wider overview of the field.
8. Discussion. The paper does not show that the theory of change has been successful in achieving outcomes as it is claimed. It shows that the interventions have been successful in specific intermediate outcomes. More explanation is needed for how the theory of change contributed to achieving the outcomes. A consideration of the limitations of this approach that the authors might have come across is also needed.
9. Reach. The study took place during the pandemic which was a unique context that led to increased student engagement with online learning more generally. Can the authors please comment on how that might have affected their results?
10. Conclusion. The conclusion refers to the theory of change as an intervention. If it is being conceptualised as an intervention, a case for this and an explanation should be provided in the introduction. The authors also conclude that the flexibility of the theory of change structure has allowed the organisation to reach its goals. What do the authors mean by flexibility? Do they really mean the interventions, rather than the theory of change itself?
11. Stakeholders (line 4 of the introduction) – please make clear stakeholders of what?
12. On first reference to LMIC please spell out what this is.
13. ‘The SRF core team realised they needed a methodology with an adequate level of operational details informed by the contextual needs to facilitate major reforms…’ (bottom of first page of introduction. I was not sure what was meant by this sentence. Please expand and explain.
14. Conceptualisation. Page numbers are needed for the direct quotations from Da Silva et al, as well as a date.
15. Formation of core group. The authors state in the text that the core group were medical students, but table 1 seems to indicate that these were people in senior positions with much experience. This is confusing, I would recommend the authors provide more explanation. As point 1 above, have all of them given their consent to be named in this way?
