# Supplementary material for: Implementation evaluation of a medical student-led intervention to enhance students’ engagement with research: Findings and lessons learned
Source: PLoS One. 2023 Aug 31;18(8):e0290867. doi: 10.1371/journal.pone.0290867 (PMC10470873; doi:10.1371/journal.pone.0290867)
Supplement: Attachment — Submitted filename: Response to Reviewers.docx [file pone.0290867.s006.docx]

February 9, 2023

Dear Editor and Reviewers,

Thank you for reviewing and critiquing our manuscript and for providing thorough comments to allow us to improve our paper. I have tabulated each comment below along with a response indicating how the authors have worked on it accordingly. Please let us know if any further revisions are required. Once again, we are grateful to the team at PLOS ONE for their consideration of our work.

Warm regards,

Mian Arsam Haroon, MBBS

Aga Khan University

[mian.haroon@scholar.aku.edu](mailto:mian.haroon@scholar.aku.edu)

| 1. Ethics. The authors have indicated that no ethical statement was needed for this study. However, in any research or evaluative study there are ethical implications for how it is carried out, even if no formal ethical approval has been sought. I had a number of concerns relating to ethics in this paper, first and foremost that it is not considered at all in the text. In addition, participants names are used, as well as organisational and role details. In and of itself, this may not be an issue, providing that permission has been sought from participants, and any unintended consequences have been considered. If this is the case, then a paragraph needs to be inserted to explain this process and the resulting ethical decisions. If not, then I would suggest anonymising any data pertaining to individuals. I would recommend referring to the British Educational Research Association (BERA) guidelines in this process as a starting point. | Thank you for the comment. The relevant information has been added to the manuscript under methods section “II. Study Design” page 7, paragraph 1. Ethical approval was sought from the Ethics Review Committee at the Aga Khan University and exemption was received (approval number 2022-7137-20351). The individuals named in this paper, along with the information on their involvement in the process, have provided their consent. Participant data is employed only in the form of numbers available from social media platforms or event attendance and is completely anonymized. |
| --- | --- |
| 2. The use of theory of change. The title implies that the paper explores the efficacy of the theory of change process. This however does not materialise and, rather, the paper explores the reach and efficacy of the interventions rather than the theory of change as an approach. I would recommend that either the title is changed to reflect this, or that more details are provided about the theory of change process itself. This needs to comprise a more detailed examination of literature concerning theory of change approaches situated in a consideration of alternative approaches that could be used. The paper then needs to explain the limitations of the approach as well as the potential benefits and provide an explanation in the conclusion about any challenges or successes that emerged from the theory of change approach. | We agree with the reviewer’s comment and have reframed the paper accordingly. We have changed the title to: ‘Implementation evaluation of a medical student-led intervention to enhance students’ engagement with research: findings and lessons learned.’ The theory of change has been reported under methods as the approach used to design the intervention activities.  The discussion has been enhanced to include limitations and potential benefits of the approach as experienced by the medical students leading the intervention. |
| 3. Interventions. It is not clear to me how the theory of change process led to the implementation of particular interventions. I would recommend considering the evidence for particular interventions in your literature review and then explaining how the adoption of a theory of change approach led to the authors (and others?) deciding these were appropriate interventions. | We have elaborated the methods section on page 10, para 2 to explain the context of why certain interventions were chosen e.g., social media as an intervention to enhance faculty engagement and recognition. The specific intervention activities were designed after discussion and mutual agreement of the core members during the design phase. Being part of the college, the core group was very aware of what the reasons were for low engagement of the stakeholders. The main guiding principle was to incentivize engagement through social media and participation of higher leadership. |
| 4. The model. I recommend giving a description of how the theory of change approach was carried out. What kinds of activities were used to develop the theory of change? How was consensus reached? How did the theory of change help in deciding what to do? | We have described development of the theory of change under methods section “III. Development of ToC”, page 7. The process for the formation of a theory of change included weekly or biweekly meetings between January to June 2019, with each meeting lasting from 45 minutes to an hour. Meetings would involve updates on a task list e.g. the evaluation and improvement of organization objectives. The fulfillment of these objectives would then be viewed through problems and obstacles for which solutions needed to be created. Progress was recorded as meeting minutes that eventually coalesced to form a primitive ToC. In the following meetings, students in the core group would present a draft of theory of change. Feedback was provided by mentors through questioning about the suggested activities and why this would encourage greater engagement. Principles of the intervention activities were based on incentive, feasibility - free courses, capability of the students, student requirements, and the efficacy of whether these strategies would be effective or not. |
| 5. Data analysis. ‘implementation outcomes were either adopted from existing literature or defined as novel and tailored interpretation’. This needs explaining and more detail given. What literature? How were they decided? | Implementation outcomes were adapted from Proctor et. al, cited as reference 18, and are defined in the following paragraphs in the data analysis section. The outcomes were decided by the core group during the design based on principles of feasibility of collecting data for the students who had to manage it along with their ongoing classes. |
| 6. Results. These are results of the evaluation based on reach, implementation and efficacy, rather than the results of using a theory of change approach and this should be made clear, and the metrics for measuring reach, adoption and efficacy clearly outlined at the beginning of the section, or in previous section around methods. The methods need to state how this information was collected, what form it was in, how it was analysed and what permissions were sought to use it. | Thank you for the comments. We have clarified in the results section that we describe the implementation evaluation of our intervention under page 27, paragraph 1.  Two authors were designated for data collection (RA, AS). The process of data collection and analysis has been described under “V. Data Acquisition and Analysis” on page 16, paragraph 3. |
| 7. I remain unsure of how Figure 1 relates to the theory of change. The outcomes for students are listed as objectives, rather than steps in the theory to enhance student engagement with research. The intermediate outcomes seem more like a list of measurements than expected changes. I would recommend reading and referring to work by Carol Weiss, and also by Mackenzie and Blamey, and then finally by Connell and Klem. Pawson’s work also provides a wider overview of the field. | Thank you for the comment. The figure has been modified as a logical framework to reflect the involvement of the interventions (listed as “activities”), steps in the process, and the desired long-term goals. The modified figure aims to accurately represent the study as a commentary on the success of the interventions instead of solely the theory of change. |
| 8. Discussion. The paper does not show that the theory of change has been successful in achieving outcomes as it is claimed. It shows that the interventions have been successful in specific intermediate outcomes. More explanation is needed for how the theory of change contributed to achieving the outcomes. A consideration of the limitations of this approach that the authors might have come across is also needed. | We agree with the comment. We have revised the manuscript to describe how the approach using theory of change has helped the students (page 42, paragraph 2). |
| 9. Reach. The study took place during the pandemic which was a unique context that led to increased student engagement with online learning more generally. Can the authors please comment on how that might have affected their results? | The COVID pandemic may have facilitated participation through means of a flexible schedule, allowing more participants to join. An increase in laptop acquisition during the pandemic could have also contributed to increased participation (page 38, paragraph 2). |
| 10. Conclusion. The conclusion refers to the theory of change as an intervention. If it is being conceptualised as an intervention, a case for this and an explanation should be provided in the introduction. The authors also conclude that the flexibility of the theory of change structure has allowed the organisation to reach its goals. What do the authors mean by flexibility? Do they really mean the interventions, rather than the theory of change itself? | These interventions are effective, but partly due to the fact that these were informed by the theory of change. The ToC was designed by a multidisciplinary team and framed by a behavioral scientist with much deliberation on *why* the proposed intervention would work. We have rephrased the conclusion that the flexibility we refer to involves the interventions and not the theory of change itself. |
| 11. Stakeholders (line 4 of the introduction) – please make clear stakeholders of what? | Stakeholders refers to medical students and has been modified appropriately. |
| 12. On first reference to LMIC please spell out what this is. | Spelled out as Low and Middle Income Countries. |
| 13. ‘The SRF core team realised they needed a methodology with an adequate level of operational details informed by the contextual needs to facilitate major reforms…’ (bottom of first page of introduction. I was not sure what was meant by this sentence. Please expand and explain. | The SRF core team realized they needed a methodology with an adequate level of operational details to facilitate major reforms needed to meet the expanded scope. The team acknowledged the lack of a set protocol to follow (page 5, paragraph 1). |
| 14. Conceptualisation. Page numbers are needed for the direct quotations from Da Silva et al, as well as a date. | Page numbers and a date has been added for the quotation from Da Silva et. al. |
| 15. Formation of core group. The authors state in the text that the core group were medical students, but table 1 seems to indicate that these were people in senior positions with much experience. This is confusing, I would recommend the authors provide more explanation. As point 1 above, have all of them given their consent to be named in this way? | The core group consisted of senior 3rd, 4th and 5th year medical students, along with faculty mentors with experience in implementation science, all of whom had been involved in research at some stage of their undergraduate medical career (Table 1). All individuals mentioned in table 1 have provided consent to be named with their roles and expertise. |
