# Supplementary material for: Implementation evaluation of a medical student-led intervention to enhance students’ engagement with research: Findings and lessons learned
Source: PLoS One. 2023 Aug 31;18(8):e0290867. doi: 10.1371/journal.pone.0290867 (PMC10470873; doi:10.1371/journal.pone.0290867)
Supplement: Attachment — Submitted filename: Response to reviewers 1.docx [file pone.0290867.s007.docx]

August 2, 2023

Dear Editor and Reviewers,

Thank you for reviewing and critiquing our manuscript and for providing thorough comments to allow us to improve our paper. I have tabulated each comment below along with a response indicating how the authors have worked on it accordingly. Please let us know if any further revisions are required. Once again, we are grateful to the team at PLOS ONE for their consideration of our work.

Warm regards,

Mian Arsam Haroon, MBBS

Aga Khan University

[mian.haroon22@alumni.aku.edu](mailto:mian.haroon22@alumni.aku.edu)

| Comment | Response |
| --- | --- |
| 1. Please update the ethics statement | The authors regretfully were not able to fully understand the reviewer’s comment. Based on the previous review and response to reviewers submitted by the authors, we have already updated the ethics statement as mentioned under methods section “II. Study Design” page 7, paragraph 1. The IRB approval for the project is attached as “ERC approval SRF ToC (1)”. Please let us know if any other changes are required. |
| 2. It is not clear whether all the of 3rd to 5th years medical students were included in the formation of core groups. The authors did state that they selected those who had been involved in research but did not explain the extent of involvement (whether completed the research module, conducted research, had publication). | We agree with the ambiguity pointed out by the reviewer. We have updated the manuscript to mention that only a few, not all, 3^rd^ to 5^th^ year medical students were involved in the formation of the core groups. We have also mentioned that the students had completed their research module and had conducted some sort of research till that point (page 8, paragraph 3). |
| 3. It is not clear how those who were involved at each stage, were recruited. | The authors have clarified in the revised manuscript that the participation of members in the core group was voluntary, given that they fulfilled the minimum requirements of research involvement in the comment #2, which all senior SRF members did (page 8, paragraph 3). |
| 4. Number of participants benefited at each intervention is not clear. e.g., Indicate number of attendees where the number of workshops is reported. | We agree with the reviewer’s comments. In the example mentioned, the authors have provided the number of attendees for certain workshops held by the Student Research Forum (page 30, paragraph 1). The number of attendees for all interventions are listed in the supporting file ‘S1 table’. Keeping in mind that the authors intended for this project to be a commentary on implementation science, for the sake of conciseness, we decided to include this information in the supporting file. If the reviewers would still prefer us to add the table to the main manuscript, the authors will gladly oblige. |
| 5. Did you document the characteristics of participants who benefitted from each intervention? | Unfortunately, we were not able to document characteristics of the participants that benefitted in our interventions. We have clarified this on page 43 in paragraph 2. |
| 6. Please include the denominator when reporting the outcomes of each intervention. | Although the authors understand the comment made by the reviewer, we regret that we are not able to provide the denominators for the outcomes. Per our viewpoint, the paper does not prioritize outcomes but aims to focus on the implementational interventions that facilitated the organization’s improvement. We see the unavailability of the denominator as twofold: 1) the denominator was too difficult to acquire e.g. the number of individuals reached out to sign up as research conference ambassadors or 2) not available e.g. the number of participants that signed up for an event but did not end up attending. |
| 7. Who facilitated the SRF? | The SRF, with respect to this paper, was facilitated by the faculty patron Babar Hasan and global health faculty Muneera Rasheed. This is mentioned on page 8 in paragraph 2 and table 1. |
| 8. Did you have the same members in the core group for the total duration of the project? | No. Over the course of this project, the number of members of the core group fluctuated. The number never went below the original 5 involved as mentioned in table 1. As members graduated from medical school, they would cease to be involved in the core group. As members were inducted into more senior roles in SRF, they would have the opportunity to be involved in the project. This has been clarified on page 13 in paragraph 1. |
